# On the Role of Seminal Fluid Protein and Nucleic Acid Content in Paternal Epigenetic Inheritance

**DOI:** 10.3390/ijms232314533

**Published:** 2022-11-22

**Authors:** Bahar Patlar

**Affiliations:** Animal Ecology, Department of Zoology, Martin-Luther University Halle-Wittenberg, 06099 Halle (Saale), Germany; bahar.patlar@zoologie.uni-halle.de

**Keywords:** seminal fluid, seminal plasma, epigenetics, transgenerational plasticity, sperm, paternal effects

## Abstract

The evidence supports the occurrence of environmentally-induced paternal epigenetic inheritance that shapes the offspring phenotype in the absence of direct or indirect paternal care and clearly demonstrates that sperm epigenetics is one of the major actors mediating these paternal effects. However, in most animals, while sperm makes up only a small portion of the seminal fluid, males also have a complex mixture of proteins, peptides, different types of small noncoding RNAs, and cell-free DNA fragments in their ejaculate. These seminal fluid contents (Sfcs) are in close contact with the reproductive cells, tissues, organs, and other molecules of both males and females during reproduction. Moreover, their production and use are adjusted in response to environmental conditions, making them potential markers of environmentally- and developmentally-induced paternal effects on the next generation(s). Although there is some intriguing evidence for Sfc-mediated paternal effects, the underlying molecular mechanisms remain poorly defined. In this review, the current evidence regarding the links between seminal fluid and environmental paternal effects and the potential pathways and mechanisms that seminal fluid may follow in mediating paternal epigenetic inheritance are discussed.

## 1. Introduction

Epigenetic mechanisms, including DNA methylation, histone modification and small non-coding RNA (sncRNA) transmission, and their functions in genome regulation and cell-to-cell transmission are not considered revolutionary topics in today’s biology research. Organisms are exposed to different external and internal environmental conditions that alter their phenotype, which is regulated by these epigenetic mechanisms during their development and lifetime. However, the heritability of epigenetically-acquired phenotypes through the transmission of the alternative functional states of these mechanisms to the next generations is a hot topic of interest [1,2,3,4,5]. Efforts are now being stepped up to develop a conceptual framework of extended heredity that takes into account the inheritance of epigenetically-acquired phenotypes [6,7,8,9,10]. We have learned that epigenetic variation just as it does in genes can also occur within populations and be transmitted to the next generations [1,11,12]. In addition, an increasing number of empirical studies have been focusing on the mechanisms of epigenetic inheritance, particularly on the role of the female and male germline [13,14,15]. Thus, I believe that these studies will soon lead to an explosion of new analytical approaches in several subjects areas of biology, such as heredity, animal conservation and breeding, and clinical research [16,17,18,19,20,21]. 

Studies on paternal epigenetic inheritance through sperm reveal the significant role of sperm epigenetic status in altering the offspring phenotype in an adaptive or maladaptive manner [22,23,24,25,26,27,28,29,30,31]. On the other hand, paternal effects through factors other than sperm epigenetics were generally assumed to be absent or less important in the absence of conventional paternal care, where the male’s role is often assumed to be limited to transmitting his DNA to the offspring [7,32]. Apart from sperm, males transfer a complex mixture of varying molecules (e.g., proteins, lipids, carbohydrates, water, etc.) and microbes in the seminal fluid (i.e., the non-sperm fraction of the ejaculate) during mating [33,34]. Notably, seminal fluid contains a number of proteins, peptides, sncRNAs, and fragments of cell-free DNA (cfDNA) that interact closely with male reproductive organs and sperm, female reproductive organs and molecules, and finally eggs and embryos. Furthermore, studies in many taxa have explicitly shown that the composition of seminal fluid (i.e., the absolute and relative amounts of proteins, peptides, etc. in the seminal fluid) responds highly plastically to different environmental factors [35,36,37,38,39,40,41,42,43,44] and is also subject to the interaction between genotypes and environments [45,46]. Therefore, seminal fluid protein, peptide and nucleic acid content (hereafter referred to as Sfc for short) may play a more significant role in epigenetic inheritance and the transmission of environmentally-induced paternal effects to the next generations than has hitherto been suspected [6,47,48,49,50,51,52]. 

In this mini-review, I focus on the evidence for seminal fluid-mediated paternal effects on offspring and discuss the proximate pathways and mechanisms related to Sfcs within the concept of *transgenerational epigenetic inheritance* (i.e., the inheritance of variations to the next generations that do not derive from differences in DNA sequence [2,53]), excluding examples from human clinical research, whereas previous reviews have addressed this more recently [54]. Note that I prefer to use the term *epigenetic inheritance* rather than *transgenerational epigenetic inheritance*, as this is a much broader term that includes mechanisms of cell-to-cell transmission as well as transmission from (grand)parents to (grand)offspring and seminal fluid can encompass both. *Epi* is a Greek prefix often used to refer to something that is “*upon*, *on*, *over*, *near*, or *at*” of something else, and is rarely used to refer to something that is “*beyond*” something else. Indeed, epigenetic mechanisms work tightly *with*, *on*, or *near* genes throughout the genome. Therefore, I have also chosen not to use the term *non-genetic* or *non-DNA inheritance* when referring to a parental effect derived from one or more of these defined epigenetic mechanisms, mainly to avoid confusion about the concept of epigenetic inheritance.

## 2. Relationships between the Seminal Fluid Content and the Phenotype of the Offspring

The supportive evidence of the link between the environmentally-induced Sfc composition of the father and the phenotype of the offspring comes from a limited number of studies in the field. In their pioneering study in the neriid fly *Telostylinus angusticollis*, Crean et al. (2014) used a so-called telegony approach, in which the female mates consecutively with two conditionally manipulated males, and the effects of the first male’s condition on the phenotype of the second male’s offspring were estimated [55]. In their experiment briefly, the females received sperm and seminal fluid from the first male raised under different nutritional conditions (either high quality or low quality) when the females were not yet fully mature. The second mating took place after the female had become sexually mature; therefore, the second male sired the majority of the offspring. They then examined and tested for the relationship between the condition of the first male and the body size of the offspring sired by the second male (i.e., step-offspring) and found that the body size was significantly affected by the condition of the first male which only suggests an effect of seminal fluid from the first male. Although they did report no direct paternal effect, the effect of seminal fluid on the body size in the subsequent generation cannot be overlooked as a potential mediator of an epigenetically-acquired phenotype, as the effect was observed in the step-offspring. 

Using a similar approach in the red flour beetle *Tribolium castaneum*, where the seminal fluid of males exposed to bacterial infection was used when one of the two males mated with the female, it was shown that the step-offspring’s immune resistance is altered by the seminal fluid of the exposed males [56]. In another study using artificial ejaculation in the European whitefish *Coregonus lavaretus*, the presence of foreign seminal fluid in the ejaculate has been shown to the increase swimming performance in the offspring [57]. The links between paternal nutrition and metabolic dysfunction in the offspring have also been repeatedly shown in mice to be mediated by either sperm-related or seminal fluid-mediated processes [58,59,60].

Embryo survival as an observed offspring phenotype has been measured in some other studies using similar experimental approach to test for paternal effects through seminal fluid. For example, variance in embryo viability is partially explained by Sfc composition [61], and seminal fluid from males on low-protein, high-carbohydrate diets reduces embryo viability [62] in the cricket *Teleogryllus oceanicus*. These results undoubtedly indicate a relationship between Sfc composition and the reproductive success of seminal fluid donors. However, caution must be exercised when interpreting these results in the context of paternal inheritance, as the population in question died before the data were collected.

Another line of studies focuses specifically on the effects of the absence of seminal fluid or its producing and storage organs. These studies often result in a lack of fertilization or embryo growth [57,63,64,65,66,67,68], but some of them have clearly shown changes in the phenotype of the offspring in the absence of seminal fluid. For example, the ablation/removal of the seminal fluid content by removing organs affected the metabolic health of male offspring in mice [69] and caused developmental and behavioral changes in the offspring of golden hamsters [70,71]. In the fruit fly *Drosophila melanogaster*, it was shown that females that received seminal fluid had daughters with higher fertility than females that did not receive seminal fluid [72]. Furthermore, when two males from different populations mated with a female of *D. melanogaster*, increased fecundity of the step-daughters was found due to the effects of the seminal fluid–maternal interactions [73]. 

Overall, the compelling evidence suggests that non-sperm fraction of the seminal fluid, which is altered by the paternal environment, can exclusively affect offspring development and phenotype. In addition, the abovementioned examples come from a variety of taxa including insects, fish, and mammals which suggests that seminal fluid-mediated paternal effects may be widespread among animals. 

## 3. The Potential Mechanisms of Seminal Fluid-Mediated Paternal Epigenetic Inheritance

**Figure 1:**
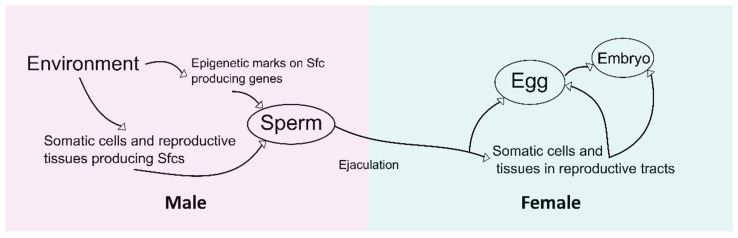
The path of seminal fluid contents (Sfcs) during reproduction. The arrows indicate the direction of environmental effects that Sfcs can be involved in during the regulation of epigenetic mechanisms to transfer the effects.

Unlike most other organic molecules, Sfcs are in close contact with various reproductive tissues and germ cells of both sexes, and eventually reach the embryos. Thus, it would not be surprising that Sfcs can have unique pathways that give them the ability to act on the epigenetic regulations of both sexes (Figure 1). In this setting, Sfcs may control environmental paternal epigenetic inheritance through at least three distinct, but likely interconnected pathways. The limited studies in the field of epigenetics research provide key ideas on the mechanisms by showing that Sfcs are capable of altering the epigenetic status of (1) sperm, (2) female reproductive tracts, and (3) eggs and embryos, as discussed below. 

### 3.1. Seminal Fluid Mediates Sperm Epigenetics

Sperm epigenetics and its role in the formation of offspring health and phenotype are now well known [23,28,29,74]. DNA methylation, histone modification, and sncRNA content of sperm are the best-established epigenetic mechanisms [22,23,53,75,76,77,78,79] that can be stable and heritable between generations [22,80,81,82]. Environmental conditions such as diet, toxins, social conditions, stress, temperature, etc., alter these epigenetic mechanisms, which are incorporated into the sperm and transmitted to the embryo, controlling and modifying the changes during embryonic development and/or in the adult life of the offspring [58,59,83,84,85,86]. Thus, considering the fact that seminal fluid is produced and used in response to such environmental conditions while in close contact with sperm during the period between meiosis and ejaculation, its contribution to epigenetic changes in sperm seems inevitable. 

First of all, there is ample evidence for a link between seminal fluid and the control of sperm RNA composition. In animals, seminal fluid carries various kinds of extracellular vesicles (e.g., micro- and nanovesicles, exosomes, proteasomes, etc.) containing Sfcs [87,88,89,90,91,92,93], and this cargo has many essential functions in sperm fertilization success [89,90,93,94,95,96,97,98,99]. The extracellular vesicles of seminal fluid can be transferred between cells and tissues and deliver the contents to a target cell/tissue [100,101,102,103]. Seminal fluid is known to be able to adjust the sncRNA composition of sperm by transferring a variety of sncRNAs within extracellular vesicles or as free molecules into the fluid [22,93,102,104,105,106,107]. In mammals, for example, the seminal vesicle and epididymis—organs where some Sfcs are produced and secreted—origin vesicles can attach to the sperm membrane and release their contents into the sperm [102,108]. Moreover, cfDNAs, mRNAs, RNAases, and double-stranded RNAs are also present in the seminal fluid [109,110] altering the sperm RNA profile [111,112,113,114]. For example, recent findings have shown that mature sperm has the permeability to take exogenous DNA [115,116,117] and has the capacity to internalize mRNA into DNA by reverse transcriptase [118]. 

The environmentally-exposed somatic cells that produce Sfcs can transmit their exposure to the germline through the transmission of extracellular vesicles or directly as vesicle-free molecules that can induce changes in the offspring [23,107,119,120,121,122]. In mice, for example, extracellular vesicles from seminal fluid that mediate sncRNAs in sperm cause a persistent transmission of paternal stress conditions that alters the transcriptome patterns in the offspring [123]. The artificial injection of testis-specific sncRNAs into the unicellular embryo has been shown to mediate the paternal effects of diet-induced obesity and metabolic disorders on the offspring [124]. Furthermore, it is known that the production of Sfcs is adjusted in many taxa depending on different environmental conditions [35,36,37,38,39,40,41,42,43,44,125,126,127]. Therefore, a novel role seems plausible for Sfcs to be messengers/markers that collect information from other tissues and transmit it to sperm embedded in different forms of nucleic acids to alter the epigenetic status of the sperm, which may ultimately shape the offspring phenotype [23,121,122].

Second, the level of Sfc-producing gene expression and/or the amount of a particular protein/peptide in the seminal fluid may also act as a signal of a male’s status and be associated with the epigenetic status of sperm. For example, the expression level of genes encoding heat shock proteins (hsp) was found to be related to changes in the morphology of *Locusta migratoria* offspring as a function of population density, and it has been suggested that hsp functions mediate paternal epigenetic regulation and maintain transgenerational plasticity [128,129]. Supporting the notion that some hsp proteins have been identified in the seminal fluid that is transferred to females in the fruit fly *D. melanogaster* [130,131], the red flour beetle *Tribolium castaneum* [132], boar [133], and humans [134], this suggests that the potential role of seminal fluid hsps in epigenetic inheritance needs to be investigated. 

Epigenetic modifications such as DNA methylation and chromatin modification in spermatozoa often occur prior to ejaculation, where spermatozoa are stored with Sfcs in the storage organs [74]. Although, the evidence is limited, there is a great potential for Sfcs to alter these epigenetic modifications in sperm. For example, the absence of glands that produce seminal fluid interferes with epigenetic reprogramming by impairing histone acetylation in the sperm of golden hamsters [70]. Exposure of seminal fluid to environmental toxins, such as those used in agriculture, has been found to be associated with hyper- or hypo-methylation of sperm DNA in humans [135,136]. Another interesting example: seminal fluid has been linked to the zinc profile of sperm chromatin, which controls chromatin instability in mammals [137,138].

On the other hand, the genes responsible for the production of Sfcs might themselves be subject to specific DNA methylations and/or histone modifications which can be heritable, as many genes are expressed at the appropriate time and levels required in male reproductive organs. For example, DNA methylation profiling of seminal vesicles, where some Sfcs are produced in mice, has shown that some toxic exposure of vesicles in early development affects their level of methylation and subsequent epigenetic reprogramming in adulthood [139]. Another very interesting study showed that cfDNAs can be a kind of courier for the DNA methylation pattern of testis and epididymis-specific gene promoters in human semen [140]. For example, the methylation level of some testis-specific promoters in cfDNA has been found to be strongly correlated with the methylation level of promoters in testicular tissues [110]. This association suggests that seminal fluid has the potential to preserve and carry the information for the DNA methylation patterns of certain genes in cfDNAs. 

As evidence has pointed out, seminal fluid can mediate sperm epigenetics through various pathways, which ultimately affects the phenotype of the offspring and therefore can be considered in the context of epigenetic inheritance. The novel idea that the paternal environment can influence the offspring through mechanisms involving information transfer between seminal fluid and sperm via Sfcs is a very promising and exciting hypothesis idea. The causes and the mechanisms of when, where and how seminal fluid composition is formed and used in the somatic cells and how it alters the epigenetics of the sperm are yet to be explained in detail. 

### 3.2. Seminal Fluid Mediates Female Epigenetics

Changes in the maternal environment can dramatically affect the phenotype of the offspring, as we have learned a lot from the outcome of research on maternal effects [141,142,143,144,145,146]. Seminal fluid is transferred to females along with sperm during mating, and its contents are capable of inducing physiological and behavioral changes in female [54,147,148,149,150,151,152]. For example, the effects of receiving seminal fluid proteins and peptides have been extensively studied in the model organism *D. melanogaster* and, studies found changes in female remating behaviors (e.g., mating receptivity), ovulation, oogenesis, sperm storage and survival, egg-laying, and a number of other reproductive processes [150,153,154,155,156,157]. Many of these seminal fluid-mediated post-mating effects on females have also been found in other species [34,132,154,158,159,160,161,162,163,164,165,166]. The seminal fluid-mediated changes in female can be considered within the scope of paternal epigenetic inheritance if they consist of at least one epigenetic mechanism at the regulation of these changes in females that can have an effect on the offspring. In humans and model mammals, intensive focus has been placed on female responses to receiving Sfcs and several specific female receptors and signaling pathways have been discovered that have been reviewed elsewhere [54,167,168]. These studies are critical for improving clinical applications as well as understanding mechanisms such as Sfc-induced gene pathways and regulation, activation and migration of immune cells, and modifications in female reproductive tissues [54,167]. Among these defined Sfc functions affecting females, Sfc-induced gene regulation particularly points to a plausible epigenetic pathway for paternal effects via females. For example, Sfcs in mammals that are transferred to the female bind to specific receptors such as TLR4 on target cells [168] and activates gene regulation leading to changes in female tissues and cells [167,169]. In boars, the exosomes present in seminal fluid have been shown to be involved in gene regulation in the uterus [169]. Females show transcriptional responses to mating [170,171], and several gene expression studies have repeatedly found evidence of Sfc-induced gene regulation in mated females in insects as well [160,170,172,173,174,175].

Among the most common genes whose expression changes in response to Sfcs are genes related to egg development, immunity, and nutrient sensing in females which suggests that Sfcs may serve as signals to impose paternal effects on offspring by controlling on gene regulation of maternal investment. For example, it has been shown that females can moderate their food intake and investment in eggs in *D. melanogaster* in response to receiving sex peptides in the seminal fluid [176,177]. Other studies in mammals have also shown that altered maternal investment in the eggs or placenta affects the phenotype of the offspring. In rodents, placental phenotypes such as weight and size were found to be altered by the absence of seminal fluid, and the modified placental composition caused metabolic disorders in the offspring [69]. In domestic animals, it has been suggested that the absence of seminal fluid during artificial insemination is likely to be related to health problems in the offspring [147,178]. Related to that, for example, the level of testosterone in the seminal fluid of the chicken *Gallus g. gallus* influences maternal investment in the eggs and ultimately the body weight of the offspring [179]. 

Overall, it is likely that the effects of Sfcs on female gene regulation altering immune and nutritional responses go far beyond the induction of successful fertilization and can change the offspring phenotype. Therefore, a male’s environment altering Sfc composition may mediate the female’s transcriptional response according to this compositional change based on the information that has come from his environment. Supporting the idea, a study has shown that the variation in transcriptional change in females is explained more by the male’s environmental background than by his genotype in bed bugs [160]. On the other hand, the mechanisms of how females respond to Sfc signals are still largely unknown especially in animals other than mammals. For example, although about 300 proteins and peptides have been listed in the seminal fluid of *D. melanogaster* [130,131], there is only one receptor, a sex peptide receptor, that has been identified that can regulate sex peptide-induced post-mating responses in multiple pathways [180,181]. There are still many unanswered questions on the role and involvement of the female as the route in modifying the phenotype of the offspring according to the information that comes from the seminal fluid of the father and the mechanisms for converting information into paternally-derived heritable signals. 

### 3.3. Seminal Fluid Mediates Egg and Embryo Epigenetics

Apart from the Sfc-mediated epigenetic changes in sperm and in female reproductive tissues, seminal fluid may also reach developing or mature oocytes and embryos and affect their epigenetic status, leading changes in the offspring phenotype. We have as of yet limited evidence of the direct fusion of Sfcs to the eggs/embryos. For example, some studies have shown that the radio-isotopically labelled amino acids of seminal fluid proteins from the ejaculate are incorporated into the ovaries and eggs of the cricket *T. oceanicus* [182], several *Drosophila* species [183], and *Anastrepha suspensa* [184]. In *D. melanogaster*, a few Sfcs of interest were also detected on the surface of eggs [185]. It is also known that Sfcs penetrate the ovaries and are involved in egg development in some species [150,152,186,187]. The direct contact of Sfcs with the egg may potentially cause epigenetic changes within the egg, considering its known role in the potential mechanisms used in altering sperm epigenetics described above. For example, in golden hamsters, disruption of organs producing Sfcs was shown to alters histone modification in fertilized eggs and the DNA methylation pattern of embryos [70]. However, the widespread occurrence of direct interactions between Sfcs and eggs, and the epigenetic reactions triggered after the contact, have yet to be clearly demonstrated. 

On the other hand, there is growing evidence that the sperm do not just pass on DNA to the eggs, but also cytoplasmic mRNA and sncRNAs that can originate from the seminal fluid [188,189]. For example, in the model organism *Caenorhabditis elegans*, it was shown that an important sperm protein, which is responsible for oocyte maturation in females, is provided by the sperm to the eggs in extracellular vesicles of seminal fluid [190]. Furthermore, the sperm-derived sncRNAs were identified to account for the 10% of embryonic RNA transferred to the egg via sperm during fertilization in *C. elegans* [191]. These sncRNAs have important epigenetic functions, such as constituting a memory of gene expression, controlling gene silencing, and regulating the antiviral immune response in subsequent generations [192,193,194,195,196]. Injection of testis-derived sncRNAs from male mice fed a high-fat diet into a unicellular embryo caused some pathological phenotypes in the adult offspring that were not observed when sncRNAs from healthy control males were injected [124]. Taken together, the evidence suggests the possibility that seminal fluid could deliver its contents to the eggs either directly by fusing into the ovaries or eggs or through the sperm which could transmit paternal environmental cues and trigger changes in the phenotype of offspring. 

## 4. Conclusions and Future Directions

The literature on paternal effects on offspring phenotype, health, and fitness is extensive [7,47,51,197], however, to my knowledge, it only constitutes a small part of the studies focused on the effects mediated exclusively by seminal fluid contents. As summarized here, studies have shown that the protein and nucleic acid contents of the seminal fluid have great potential to be involved in the regulation of epigenetic mechanisms in the pathway between spermatogenesis and early embryogenesis. We are only at the beginning of our understanding of seminal fluid-mediated paternal epigenetic inheritance. It is clear that further research is needed to understand how the environmental conditions of the father can contribute to heritable information in the seminal fluid. It will therefore be of interest to determine the general occurrence of Sfc-mediated paternal epigenetic inheritance in different taxa and under different environmental conditions. In addition, further studies are needed covering a wide range of phenotypes in the offspring including the highly plastic ones such as life history traits, social and mating behaviors, etc., which will allow for a better understanding of the pathways of epigenetic mechanisms in which seminal fluid may be involved. Therefore, studies with high-throughput profiling of proteins, peptides, mRNAs, sncRNAs, and cfDNAs in the seminal fluid under e different environmental conditions are needed to test the links between offspring phenotypes and Sfc composition. In addition, comprehensive studies on the occurrence, pattern, and inheritance of DNA methylation or other modifications during gene regulation under different conditions in reproductive tissues producing Sfcs are also needed to better elucidate the mechanisms of Sfc-mediated paternal epigenetic inheritance. 

To estimate the paternal effects mediated by Sfc and to define the mechanisms exclusively, experiments must be carefully designed because of the handicap of sperm and seminal fluid integrity. First, experimental set-ups must disentangle the paternal effects that occur through seminal fluid that arise independently of sperm. The use of animal models, such as genetically-modified males that can only produce and transfer seminal fluid can help to control sperm-borne genetic or epigenetic effects on offspring. For example, conducting a double-mating assay where a female mates with two males in turn, but receives sperm from only one of them, could help to unravel the sources of phenotypic variance in offspring explained by seminal fluid or sperm-borne factors. In addition, the confounding genetic effects of both sexes, and their interactions must also be controlled while testing for Sfc-mediated paternal effects, especially in light of the evidence for paternal genetically-driven maternal effects. If an effect on the phenotype of the offspring is observed when only the seminal fluid composition is manipulated while all other sources of variation are held constant, then it can be assumed that the effect is caused by Sfcs. To control for such effects while estimating environmental paternal effects on offspring phenotype, a quantitative epigenetic approach can be very useful to improve the study design that has been recently highlighted [19,20,198].

Given their role in reproduction, the importance of Sfcs has recently been highlighted in the context of assisted reproductive technology and has already begun to improve the process of in vitro fertilization in humans [147,199] and in domestic animals [147]. Going forward, a deeper understanding of the role and mechanisms of seminal fluid and the associated environmental factors on paternal epigenetic inheritance may be critical to advancing these fields, as well as others such as conservation ecology. If selection favors an environmentally-induced seminal fluid-mediated phenotype in offspring that maximizes advantages, this could therefore be evolutionary adaptive. In this context, it is crucial to understand the non-adaptive and adaptive consequences of the changing composition of seminal fluid in response to environmental conditions. For example, rising temperatures may changes in seminal fluid composition which may change population persistence to global climate change. On the other hand, in the industry of animal husbandry, the traditional approach to improve artificial selection regimes solely relies on genetic variability, while the effects of paternal conditions are often ignored. Knowing more about the functions of Sfcs in mediating environmental paternal effects can also have implications in improving farming conditions and breeding regimes when taking the environmental conditions of male brood stock on farms into account.
